# Aging-related features predict prognosis and immunotherapy efficacy in hepatocellular carcinoma

**DOI:** 10.3389/fimmu.2022.951459

**Published:** 2022-09-15

**Authors:** Ting Hong, Wei Su, Yitong Pan, Chenxi Tian, Guang Lei

**Affiliations:** ^1^ Department of Radiation Oncology, Hunan Cancer Hospital and the Affiliated Cancer Hospital of Xiangya School of Medicine, Central South University, Changsha, China; ^2^ Department of Gynecology Oncology, Hunan Cancer Hospital and The Affiliated Cancer Hospital of Xiangya School of Medicine, Central South University, Changsha, China; ^3^ Department of Medical Oncology, Fudan University Shanghai Cancer Center, Shanghai, China; ^4^ Key Laboratory of Genomic and Precision Medicine, Beijing Institute of Genomics, Chinese Academy of Sciences (CAS), Beijing, China

**Keywords:** aging microenvironment subtypes, hepatocellular carcinoma (HCC), immunological therapy, CTNNB1 mutation, prognostic model

## Abstract

The aging microenvironment serves important roles in cancers. However, most studies focus on circumscribed hot spots such as immunity and metabolism. Thus, it is well ignored that the aging microenvironment contributes to the proliferation of tumor. Herein, we established three prognosis-distinctive aging microenvironment subtypes, including AME1, AME2, and AME3, based on aging-related genes and characterized them with “Immune Exclusion,” “Immune Infiltration,” and “Immune Intermediate” features separately. AME2-subtype tumors were characterized by specific activation of immune cells and were most likely to be sensitive to immunotherapy. AME1-subtype tumors were characterized by inhibition of immune cells with high proportion of Catenin Beta 1 (CTNNB1) mutation, which was more likely to be insensitive to immunotherapy. Furthermore, we found that CTNNB1 may inhibit the expression of C-C Motif Chemokine Ligand 19 (*CCL19*), thus restraining immune cells and attenuating the sensitivity to immunotherapy. Finally, we also established a robust aging prognostic model to predict the prognosis of patients with hepatocellular carcinoma. Overall, this research promotes a comprehensive understanding about the aging microenvironment and immunity in hepatocellular carcinoma and may provide potential therapeutic targets for immunotherapy.

## Introduction

Aging is the single most robust risk factor for cancer incidences, with more than 60% of cancers occurring in those aged over 60. Cancer ranked first in the cause of death in people aged 60–79 years (1). Aging also predicts cancer prognosis. Older people have unfavorable outcomes when diagnosed with cancer (2, 3). The number of people aged over 60 is set to double by 2050 based on the World Health Organization, which puts forward cancer as an increasingly major cause of death and attracts more attention to understand the relationship between aging and cancer.

The mechanism behind the role of aging in the progression of cancer is being investigated but largely remains unclear. Both cancer and aging depend on the accumulation of cellular damage. Consequently, many hallmarks are shared between cancer and aging, including cellular senescence, microenvironmental changes, and epigenetic reprogramming. One key point of the cancer-inducing feature of aging is that aging can drastically change the tumor microenvironment by affecting various normal cells, which act to promote tumorigenesis and metastasis. Among the normal cells, immune cells appear to be more susceptible in the process of aging. For example, the immune system becomes dysregulated as people age, including the decline of the function of effector immune cells and overall immune and combined with the activation and infiltration of immunosuppressive cell populations (4). Despite the important relationship between the aging microenvironment and cancer, the function of the aging microenvironment in therapy response is often poorly represented in clinical studies. To be specific, only 40% of patients enrolled in cancer clinical trials are over 65 and less than 10% are over 75 years of age (4). Aged mice have been applied to preclinical immuno-oncology research to represent declining immune cell function and kidney more faithfully (5, 6). Yet, most preclinical studies used 8-week-old mice rather than senior mice (4). Thus, it is largely unclear how the aging microenvironment could affect immunotherapy efficacies.

The aggressiveness of cancers arising from different organs appears to correlate differently with age. Thus, the aged local and systemic environments both need to be studied. Among the different organs, liver, as an important immune and metabolic organ, is also greatly affected by aging in terms of its function and structure, which involves the regulation of the development of liver hepatocellular carcinoma (LIHC) *via* liver microenvironment modulation (7).

Thus, understanding the function of the aging microenvironment in cancers can provide meaningful insights into immunological therapy regimens and the discovery of potential therapeutic targets. Here, we established three pan-cancer conserved subtypes and uncovered their features in multiple omics. We focused on LIHC and found that the mutation of Catenin Beta 1 (CTNNB1) may inhibit the expression of C-C Motif Chemokine Ligand 19 (*CCL19*), thus restraining immune cells and attenuating the sensitivity to immunotherapy. In addition, we also uncovered the phosphatidylinositol-3-kinase (PI3K)/Akt and the mammalian target of rapamycin (mTOR) signaling pathway, which can be activated by multiple aging-related genes including *EEF1E1* and *HDAC2* and may be a potential pathway for explaining how aging-related genes affect liver tumor progression.

## Methods

### Acquisition of data

Transcriptome data and somatic mutation matrices of 14 types of cancer were collected from the University of California Santa Cruz (UCSC) database (https://xena.ucsc.edu/) (8). Meanwhile, corresponding clinical data were also obtained (**Supplementary Table S3**). The list of 500 aging-related messenger RNAs (mRNAs) was downloaded from the Aging Atlas database (9) (https://ngdc.cncb.ac.cn/aging/index) (**Supplementary Table S1**). To validate the conservatism of this subtype, additional independent LIHC validation sets, GSE76427 (10), GSE14520 (11), and GSE54236 (12), containing 167, 43, and 161 samples, respectively, were obtained from the Gene Expression Omnibus (GEO) database (https://www.ncbi.nlm.nih.gov/geo/). In addition, we also downloaded single-cell dataset, GSE149614 (13), to investigate the function of aging-related genes in the tumor microenvironment. In order to verify the expression of *CCL19* in CTNNB1 mutation set and CTNNB1 wild-type set, we also downloaded the transcriptome data and corresponding clinical information of GSE9829 (14). In addition, to verify the credibility of the prognostic model, we downloaded the transcriptome data and corresponding clinical information of GSE20140 (15) from the GEO database.

### Identification of recurrently mutated genes

For the purpose of identifying recurrent driver genes, we involved MutSig2CV (16), a tool that needs to be performed in MATLAB. In this analysis, MutSig2CV performs three tests of significance for each gene based on abundance (mutation rate), cluster (physical co-location of mutations), and evolutionary conservation. P value <0.05 and q value <0.05 were used as the criteria for screening recurrently mutated genes.

### Assessment of immune infiltrating cells

To delineate the association between subtypes and immunity, we downloaded the infiltration estimation matrices of immune associated cells of patients with LIHC, which were calculated by CIBERSORT (17), XCELL (18), TIMER (19), and MCPCOUNTER (20) algorithms, respectively, from TIMER2.0 database.

### Assessment of pathway activity by using PROGENy

PROGENy (21), a method that can infer pathway activity from gene expression by using core pathway-responsive genes, which were computed by leveraging a large compendium of publicly available perturbation experiments, was involved to calculate the activity of various pathways of LIHC patients, including the Androgen, Estrogen, epidermal growth factor receptor(EGFR), Hypoxia, Janus kinase-signal transducers and activators of transcription (JAK-STAT), mitogen-activated protein kinases (MAPK), nuclear factor-κB (NFκB), PI3K, protein 53 (p53), transforming growth factor β (TGFβ), Tumor necrosis factor α (TNFα), tumour necrosis factor related apoptosis-inducing ligand (TRAIL), Vascular endothelial growth factor (VEGF), and Wingless-Type (WNT) pathways.

### Gene function enrichment analysis using gene set enrichment analysis

In order to probe the biological pathways associated with aging-related genes in the model, gene set enrichment analysis (GSEA), a knowledge-based approach for interpreting genome-wide expression profiles, was performed in the high-expression and the low-expression groups compared with the median level of gene expression (22).

### Assessment of protein level by using the Human Protein Atlas database

The Human Protein Atlas (23) (HPA; https://www.proteinatlas.org/) database was used to explore the protein level of *EEF1E1* and *HDAC2* in hepatocellular carcinoma and normal tissue samples. In addition, we collected protein expression labeling information (weak/moderate/high) from the HPA website of EEF1E1 and HDAC2 in immunohistochemical figures and calculated the significance between the number of normal tissue samples and hepatocellular carcinoma samples in protein expression level by using the chi-square test.

### Single-cell data processing and cluster annotation

Seurat v.3.0.0 (24) was involved for data normalization, dimensionality reduction, and clustering using default parameters. In preprocessing, cells are filtered based on the criteria of expressing a minimum of 200 genes. Also, cells that had more than 20% expression on mitochondrial genes were also removed. Cell clusters were annotated using singleR (25) with classical cell signatures.

### Western blotting analysis

The total cellular proteins from each group were extracted using Radio Immunoprecipitation Assay (RIPA) lysis buffer with 1% phenylmethanesulfonyl fluoride (PMSF). Then, equal amounts (20 μg) of protein determined by Bicinchoninic Acid (BCA) protein assay kit (Thermo Fisher Scientific, Waltham, MA, USA) were separated using 10% sodium dodecyl sulfate polyacrylamide gel electrophoresis (SDS-PAGE) gels. The proteins were then transferred to polyvinylidene difluoride(PVDF) membranes (0.45 mm, Solarbio, Beijing, China). The membranes were blocked with 5% nonfat milk for 1 h at room temperature and then incubated with primary antibodies at 4°C for 12 h. The following antibodies were tested: Histone Deacetylase 2(HDAC2), glyceraldehyde-3-phosphate dehydrogenase(GAPDH) (1:1,000; Proteintech Group Inc.) rabbit polyclonal antibodies. The secondary antibodies were anti-mouse or anti-rabbit antibody and conjugated to horseradish peroxidase (HRP) (1:4,000; Proteintech Group Inc.). The secondary antibodies were used at a 1:4,000 dilution and were incubated for approximately 1 h at room temperature. The bands were visualized with Enhanced chemiluminescence (ECL) reagents (Thermo Fisher Scientific) and developed by Omega Lum G (Aplegen).

### RNA extraction and Reverse transcription quantitative PCR

Total RNA was extracted by TRIzol Reagent (Invitrogen) from cells. Complementary DNA (cDNA) was obtained from total RNA with PrimeScript™ RT reagent kit (Takara Bio, Inc., Otsu, Japan). mRNA expression was assessed by real-time quantitative PCR (RT-qPCR), which was carried out in triplicate by a SYBR Premix Ex Taq™ kit (Takara Bio) and ABI 7900HT Real-Time PCR system (Applied Biosystems Life Technologies, Foster City, CA, USA). The primers used are shown in **Supplementary Table S3**.

### Immunohistochemistry

Secondary liver cancer samples were collected, embedded, and sectioned. Immunohistochemical staining was performed according to standard protocol, and the primary antibody was anti-HDAC2 (1:100, Proteintech). The antibody catalog in the Western blot and immunohistochemistry (IHC) analysis is 12922-3-AP (Proteintech Group Inc.). The staining scores were calculated using the immunoreactive score (IRS) system. The percentage of positive cells was scored as follows: no stained cells = 0; <10% staining = 1; 10%–50% staining = 2; 51%–80% staining = 3; and >80% staining = 4. The staining intensity was scored as follows: no color reaction = 0; mild reaction = 1; moderate reaction = 2; and intense reaction = 3. The IRS scores = (scores of staining intensity) × (scores of percentage of positive cells).

### Statistical analysis

According to the aging-related genes that were downloaded from the Aging Atlas database (9), we divided LIHC patients into three groups by applying R package “CancerSubtypes” (26). The survival rate of each subtype was calculated by using the Kaplan–Meier (K-M) method, and the log-rank test was used to assess the difference in survival among the three subtypes with a criterion level of P < 0.05. Wilcoxon test and Kruskal–Wallis test were involved to estimate the subtype-specific differences and the mRNA expression differences related to other factors. Briefly, when clinical traits have two characteristics, Wilcoxon test was involved to examine the significance. Meanwhile, Kruskal–Wallis test was applied when the clinical features have more than two characteristics. “Limma” (27) package was used to calculate the marker genes of each subtype. Furthermore, hierarchical cluster analysis was performed to classify the validation set *via* the ward.D method. In addition, the difference between the proportion of clinical factors and subtypes was computed by the chi-square test. Search Tool for the Retrieval of Interaction Gene (STRING) database was applied to calculate the relationship between marker genes of each subtype, and Cytoscape was involved to describe the network of these genes. The relationships between the expression of mRNAs and immune infiltrating cells were calculated by Spearman’s correlation coefficient. Aging-related mRNAs significantly associated with survival were identified by using univariate Cox proportional hazards regression. Then, least absolute shrinkage and selection operator (LASSO) regression analysis and multivariate Cox regression was applied to establish the aging-related prognosis model based on aging-related genes. Multivariate Cox regression was also used to assess the independence of the model compared with other clinical factors such as Grade, T stage, and Stage. Also, the credibility and predictive value of our model were evaluated through time-related receiver operating characteristic (ROC) curve. Moreover, waterfall plot was created by using the maftools (28) package. All statistical analyses were performed using R-version 4.1.1.

## Results

### Liver cancer was characterized by a unique aging microenvironment by pan-cancer analysis and can be divided into three subtypes

**Figure 1:**
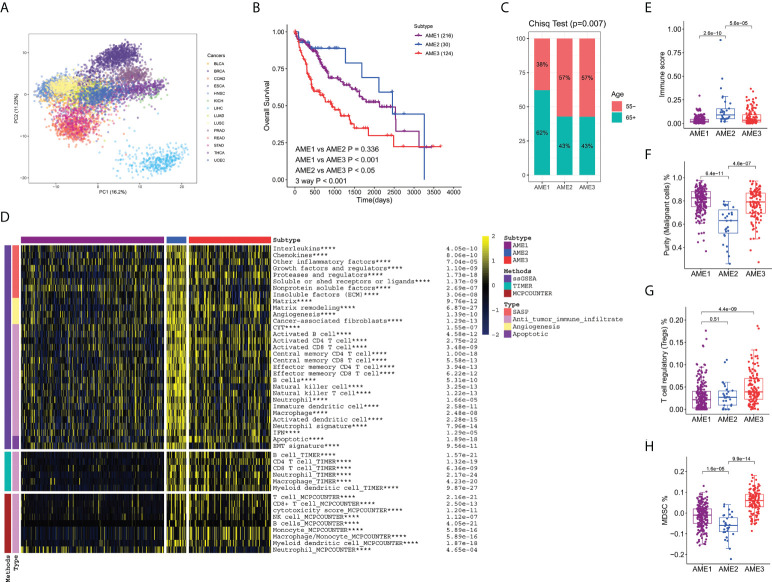
Identification of three prognosis-distinct aging microenvironment subtypes. **(A)** Principal Component Analysis (PCA) plot of 14 cancer types from TCGA. Based on the first two PCs of PCA, LIHC was characterized by a unique expression pattern of aging-related genes, which was completely different from those of other cancer types. **(B)** Kaplan–Meier estimates of overall survival of the three aging microenvironment subtypes. AME2 patients had the highest survival rate, whereas patients in the AME3 subtype had the worst prognosis outcomes. **(C)** The proportion of different ages of patients in the three subtypes. **(D)** Heatmap of immune features in subtypes, calculated by single sample gene set enrichment analysis (ssGSEA), Tumor Immune Estimation Resource (TIMER), and Microenvironment Cell Populations-counter(MCPCOUNTER). **** P<0.0001). **(E)** Boxplots of the immune score in the subtypes. **(F)** Boxplots of the purity of the tumor cells in the subtypes. **(G)** Boxplots of the proportion of regulatory T cells (Tregs) in the subtypes. **(H)** Boxplots of the proportion of Myeloid-derived suppressor cells (MDSCs) in the subtypes. Kruskal–Wallis test was performed in panels **E–H**, and P values are shown.

Principal component analysis (PCA) was applied to detect the expression patterns of aging-related mRNAs in 14 types of cancer containing LIHC from The Cancer Genome Atlas(TCGA). Based on the first two principal components (PCs) of PCA, LIHC was characterized by a unique expression pattern of aging-related genes, which was completely segregated from other cancer types (**Figure 1A**). Thus, patients with LIHC may have unique expression patterns of aging-related genes. In order to characterize the heterogeneity in patients with LIHC, CancerSubtypes, a method of classing, was implied to identify the molecular subtypes of LIHC patients. As a result, LIHC patients were divided into three aging microenvironment (AME) subtypes (AME_I_ [I = {1,2,3}]) with completely different prognosis characteristics (**Figure 1B**, **Supplementary Table S1**). AME2 patients had the highest survival rate, whereas patients in AME3 subtype had the worst outcomes. To uncover the characteristics of age in these subtypes, we defined patients older than 65 as senility and those younger than 55 as youth. Using the chi-square test, we found that patients in the AME1 subtype had the highest proportion of senility (**Figure 1C**).

### The aging microenvironment subtypes showing noteworthy differences in immune features

For the purpose of characterizing the molecular basis of these three aging microenvironment subtypes in LIHC, we downloaded the infiltration estimation matrix of immune cells in patients with LIHC, which were calculated by TIMER and MCPCOUNTER and so on, from TIMER2.0 database (http://timer.cistrome.org/). Using different algorithms, we found a consistent result that in the AME1 subtype, almost all types of immune infiltrating cells such as CD8 T cells, CD4 T cells, and Natural Killer (NK) cells were significantly suppressed, whereas samples in the AME2 subtype showed high infiltration of immune cells (**Figure 1D**). Meanwhile, the AME3 group was characterized by intermediate feature. Thus, we characterized AME1 as “Immune Exclusion” feature, AME2 as “Immune Infiltration” feature, and AME3 as “Immune Intermediate” feature, which were exactly consistent with the results of previous studies (29). Interestingly, the immune score, calculated by XCELL, also significantly activated in the AME2 subtype (**Figure 1E**). Also, immune infiltration cells, related to the inhibition of tumor cells, including Macrophage, B cell, CD4 T cell, CD8 T cell, and NK cell, were significantly activated in the AME2 subtype (**Figure 1D**). Also, the purity of tumor cells was significantly decreased in AME2 (**Figure 1F**). Nevertheless, immune infiltration cells, related to the proportion of tumor cells, just like regulatory T cells (Tregs) and myeloid-derived suppressor cells (MDSCs), were noteworthy suppressed in the AME2 subtype (**Figures 1G, H**). This result was consistent with the highest survival rate of the AME2 subtype.

### Catenin Beta 1 (CTNNB1) may be responsible for the characteristics of immune exclusion in patients with the AME1 subtype

**Figure 2:**
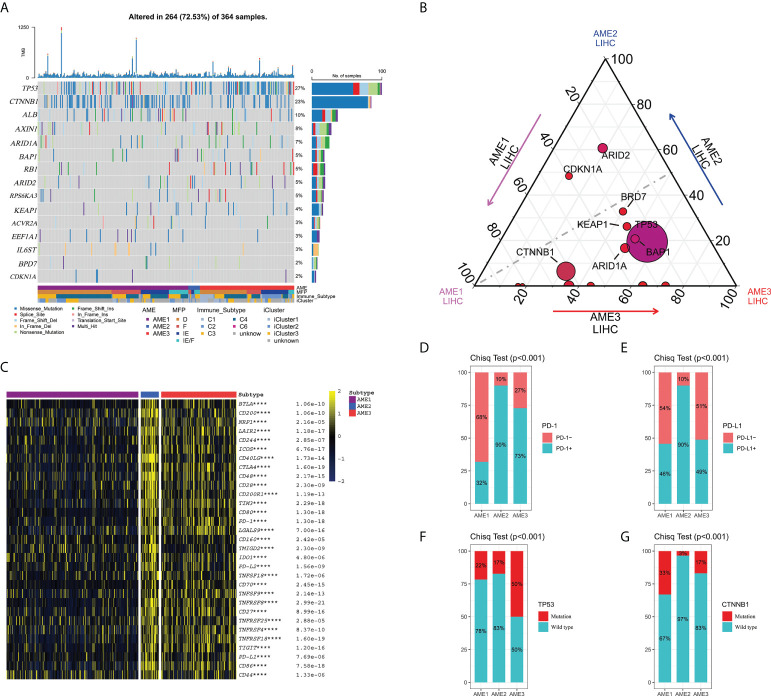
Identification of mutation characteristics and the expression of checkpoints in the different AME subtypes. **(A)** Waterfall plot of the top 12 recurrently mutated genes in LIHC, which was calculated by MutSig2CV. **(B)** The mutation number of the 12 genes in each subtype. The size of the circle represents the proportion of mutated tumors in all LIHC tumors, and the distance of the gene to a subtype on a corner represents the proportion of mutated tumors in that subtype. **(C)** Heatmap of the expression of immune checkpoints in the subtypes. ****P < 0.0001) **(D)** The proportion of PD1+ and PD1- in the subtypes. **(E)** The proportion of PD-L1+ and PD-L1- in the subtypes. **(F)** The proportion of the mutation number of TP53 in the subtypes. **(G)** The proportion of the mutation number of CTNNB1 in the subtypes.

Somatic mutation, a molecular characteristic associated with aging, has also been proven to be associated with immune cells (30). By applying MutSig2CV, we identified 15 recurrently mutated genes (q < 0.05), 12 of which were found in at least 10 patient samples (**Figure 2A**, **Supplementary Table S1**). The top putative candidate drivers from these patients with LIHC were Tumor Protein P53 (*TP53*), AT-Rich Interaction Domain 2 (*ARID2*), BRCA1-Associated Protein 1 (*BAP1*), Albumin (*ALB*), RB Transcriptional Corepressor 1 (*RB1*), Axis Inhibition Protein 1 (*AXIN1*), Kelch-Like ECH-Associated Protein 1 (*KEAP1*), Bromodomain-Containing Protein 7 (*BRD7*), Ribosomal Protein S6 Kinase A3 (*RPS6KA3*), Activin A Receptor Type 2A (*ACVR2A*) (**Figure 2A**). Specifically, the mutation proportion of *CTNNB1* was the highest in the AME1 subtype, the mutation proportion of *TP53* and *BAP1* was the highest in the AME3 subtype, and the mutation proportion of *ARID2* was the highest in the AME2 subtype (**Figure 2B**).

Immune checkpoints, as primary immune therapeutic targets, played important roles in the human immune system and have been widely applied in clinical treatment, which has achieved good clinical outcomes (29, 31). Therefore, we investigated the expression characteristics of immune checkpoints in these three subtypes. According to the expression of immune checkpoints, we find that almost all immune checkpoint expression levels were significantly higher in the AME2 subtype than those in the other two subtypes, containing Cytotoxic T Lymphocyte-Associated Protein 4 (*CTLA4*), T-Cell Immunoglobulin Mucin 3 (*TIM3*), Programmed Cell Death 1 (*PD-1*), Indoleamine 2,3-Dioxygenase 1 (*IDO1*), Programmed Cell Death 1 Ligand 2 (*PD-L2*), T-Cell Immunoreceptor with Ig and ITIM domains (*TIGIT*), and Programmed Cell Death 1 Ligand 1 (*PD-L1*) (**Figure 2C**). However, the expression of *PD-1/PD-L1* was significantly restrained in the AME1 subtype, which was in accordance with previous research. On the contrary, the expression levels of *PD1/PD-L1* and T cells in the AME2 subtype were significantly increased (**Figures 2D**, **E**). These results indicated that patients within the immune infiltration subtype may most likely respond to immune checkpoint inhibitors (ICIs), while those within the immune exclusion subtype might have innate resistance to anti-PD1/PD-L1 or similar therapies (32). In addition, we also explored the association between the mutation of *TP53* and *CTNNB1* and clinical factors including Stage, T, M, N, and status. Consequently, we found that there was no significant association between *TP53* and *CTNNB1* mutation and clinical factors (**Supplementary Figures S1A**, **S1B**). Moreover, we also demonstrated the amino acid mutation sites of TP53 and CTNNB1 (**Supplementary Figures S1C**, **S1D**). By applying chi-square test, we found that the mutation rate of *TP53* and *CTNNB1* had significant differences among the three subtypes (P < 0.05), among which *TP53* mutation rate was dramatically increased in the AME3 subtype (**Figure 2F**), while the *CTNNB1* mutation rate was significantly increased in the AME1 subtype (**Figure 2G**). In agreement with previous research, the effects of PD-1/PD-L1 ICI therapy in LIHC patients with *CTNNB1* mutation were very poor (32). This suggests that CTNNB1 may be responsible for the characteristics of immune exclusion in patients with the AME1 subtype.

### The aging microenvironment subtypes are conserved across multiple cancers and prognosticate better than other subtyping methods

Many previous classification methods failed to classify independent datasets other than the training datasets. We used the aging-related gene signatures in a pipeline that could perform pan-cancer classification. We designed our pipeline using supervised clustering. This pipeline can be divided into five steps: 1) mRNA expression profile between the AME_j_ (j={1,2,3}) group and other subtypes AME_m_ (m={1,2,3} & m!=j) and AME_k_ (k={1,2,3} & (k!=j) & (k!=m)) was compared by using limma package. 2) |LogFC| >0 and False discovery rate (FDR) <0.05 were used as the criteria for screening differentially expressed aging-related mRNAs. 3) Duplication genes that were specifically upregulated in each subtype were removed from each group. Then, the top 50 aging-related genes in each subtype were selected as markers in each group according to their logFC. 4) Then, samples were divided into three subtypes through hierarchical clustering, among which “ward.D” was selected as the method.

**Figure 3:**
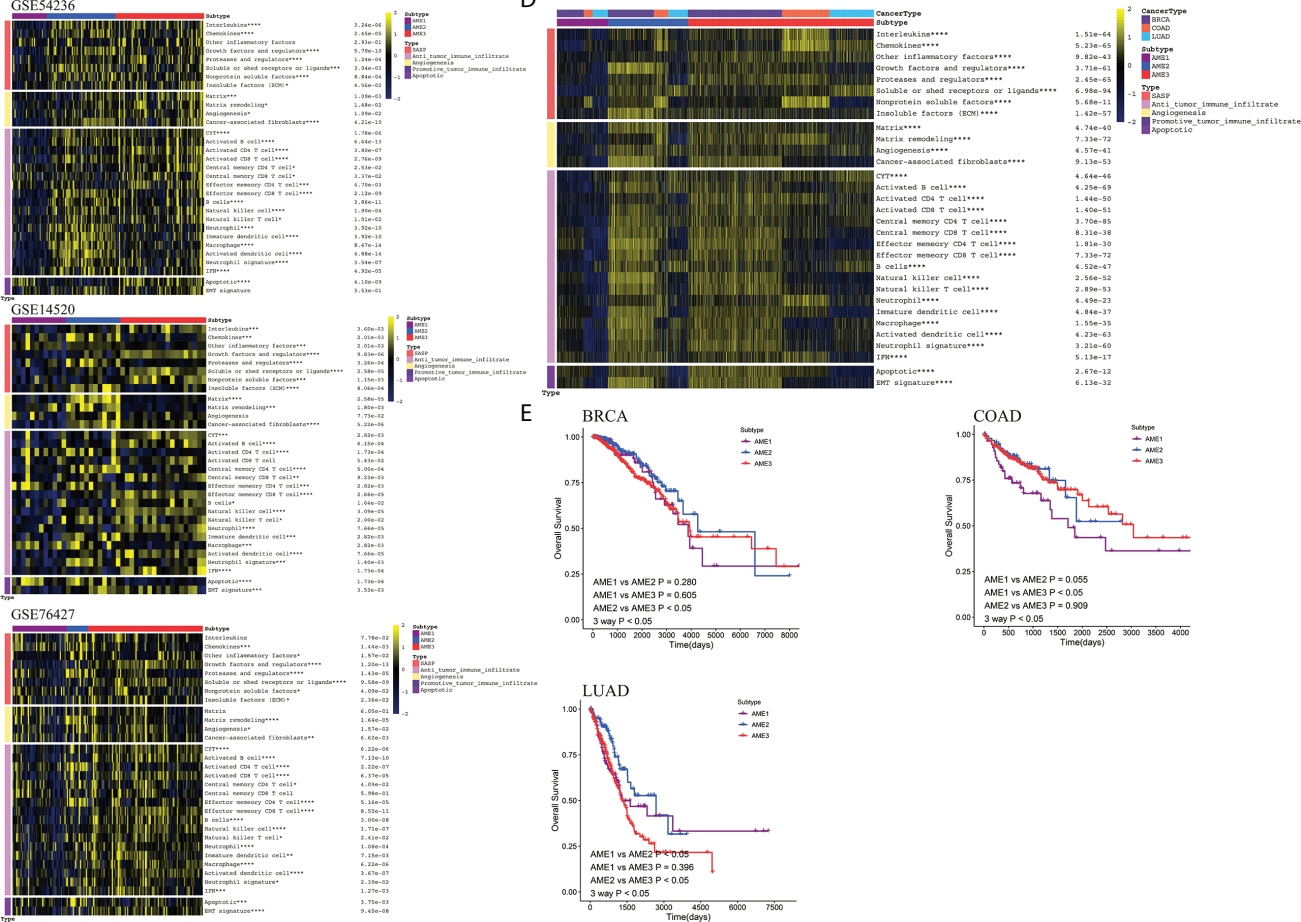
Validation of the conservation of subtypes in datasets from multiple types of cancer. **(A–C)** Heatmap of the features in different LIHC datasets from GEO (GSE14520, GSE54236, GSE76427), showing that the different subtypes shared similar features. (*P < 0.05; **P < 0.01; ***P < 0.001; ****P < 0.0001) **(D)** Heatmap of the features in different cancers from TCGA (BRCA, COAD, LUAD), showing that the different subtypes shared similar features. **(E)** Kaplan–Meier estimates of patient overall survival categorized by the three AME subtypes in BRCA, COAD, and LUAD patients.

We calculated each subtype’s markers using the pipeline shown above (**Supplementary Figures S2A**-**S2C**, **Supplementary Table S1**). Moreover, we also described the correlation between these markers by applying Spearman correlation coefficient. As shown in **Supplementary Figure S2D**, these markers can be divided into three groups, which suggested that the markers of each subtype were interrelated. Moreover, we also investigated the protein–protein intersection network between these markers by using STRING software (**Supplementary Figure S2E**). Then, three independent validations of LIHC transcriptomic data (GEO: GSE76427, GSE14520, GSE54236) were analyzed to determine whether our subtypes were conserved. In accordance with the classification characteristics of our subtypes, the three groups of GEO samples were divided into three subtypes: AME1, AME2, and AME3, indicating that the expression signature based on the aging-related genes can be applied to independent LIHC samples, among which the immune signatures in the AME2 subtype were higher than that in the AME1 and AME3 subtypes, and other features such as the Epithelial–mesenchymal transition (EMT) signature were not significant in the three subtypes (**Figures 3A–C**, **Supplementary Table S1**). In addition, by applying the pipeline, three additional types of tumors [Colon adenocarcinoma (COAD), Breast invasive carcinoma (BRCA), Lung adenocarcinoma (LUAD] in TCGA were classified into the three subtypes (**Figure 3D**, **Supplementary Table S1**), which had significant correlation to overall survival (OS) (P < 0.05), indicating that this classification pipeline can be broadly applied at the pan-cancer level (**Figure 3E**).

**Figure 4:**
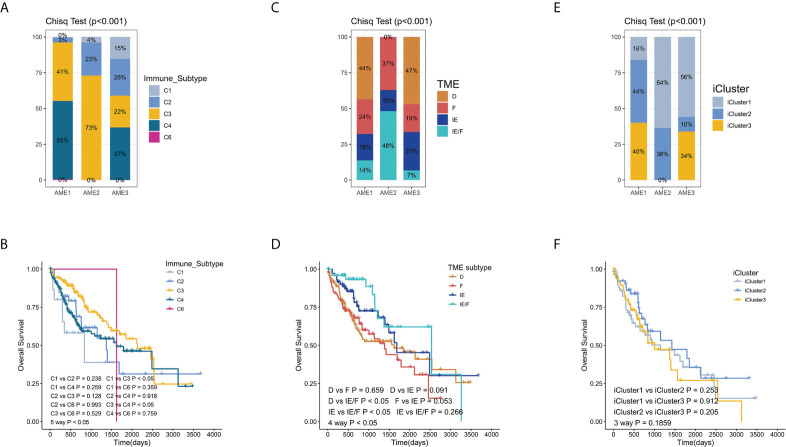
Comparing the AME subtypes to other classification methods. **(A–C)** The proportion of other clusters in each aging microenvironment subtypes. **(D–F)** Kaplan–Meier estimates of overall survival of patients with other classification methods (TME subtype, Immune subtypes, and iCluster).

We next compared our subtyping method with three other classification methods including TME subtype (33), Immune-Subtype (34), and iCluster (35) in TCGA-LIHC samples (**Figures 4A, C, E**; **Supplementary Table S1**). The proportion of C3 subtype is the largest in AME2, while the proportion of the C1 subtype is the largest in AME3. Accordingly, the C3 subtype has the highest survival rate and the C1 subtype has the lowest survival rate, which are consistent with the characteristics of the AME2 subtype and AME3 subtype, respectively (**Figures 4A, B**). When analyzed within the two subtypes separately, our AME classification model exhibited more significant correlation with OS than that in the Immune-Subtype method (**Figures 1B**, **4B**). Consistent with the previous results, the proportion of IE (Immune-Enriched Fibrotic) subtype and IE/F (Immune-Enriched, Non-Fibrotic) subtype was the highest in AME2, which further confirmed that AME2 is characterized by immune infiltration. Meanwhile, the survival rates of the IE subtype and IE/F subtype were significantly higher than those of D (Depleted) subtype and F (Fibrotic) subtype (**Figure 4C**). When compared to our AME classification model, TME subtypes have poor survival segregation in LIHC (**Figures 1B**, **4D**). Lastly, survival rates among iCluster subtypes were not statistically different (**Figure 4F**).

**Figure 5:**
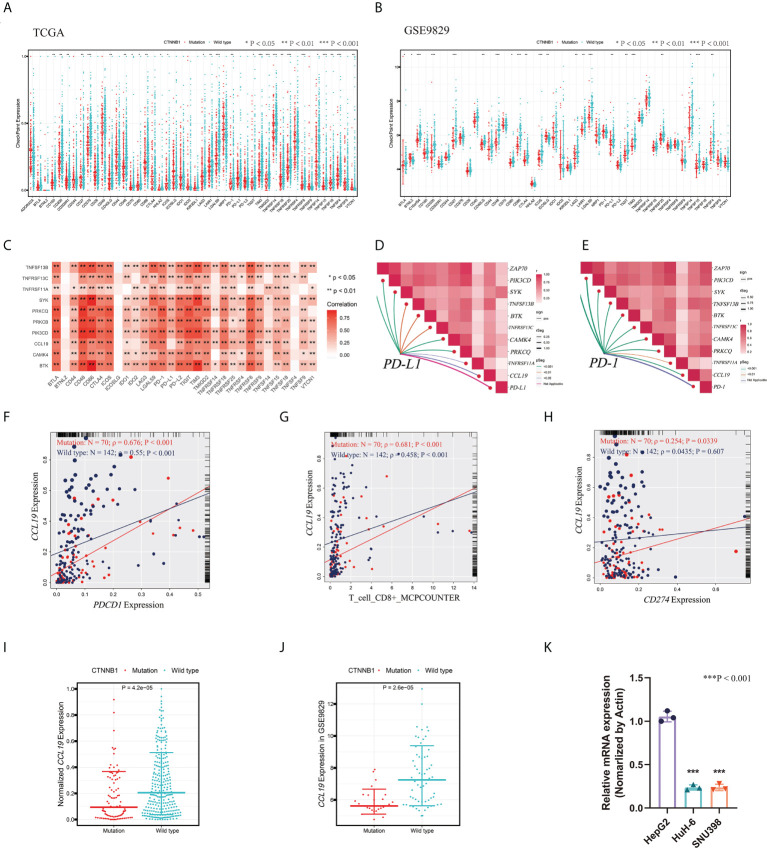
Identification of the function and the mechanism of CTNNB1 in the AME1 subtype. **(A)** Boxplots of the expression of immune checkpoints in the CTNNB1-Mutation group and CTNNB1-Wild type group in TCGA LIHC samples. **(B)** Boxplots of the expression of immune checkpoints in the CTNNB1-Mutation group and CTNNB1-Wild type group in the GSE9829 dataset (*P < 0.05; **P < 0.01; ***P < 0.001). **(C)** Spearman’s correlation of immune checkpoints and the 11 aging-related genes. **(D)** Spearman’s correlation of PD-L1 and the 11 aging-related genes. **(E)** Spearman’s correlation of PD1 and the 11 aging-related genes. **(F)** Spearman’s correlation of *PD1* and *CCL19* in the CTNNB1 mutation and CTNNB1 wild type samples. **(G)** Spearman’s correlation of PD-L1 and *CCL19* in the CTNNB1 mutation and CTNNB1 wild type samples. **(H)** Spearman’s correlation of *PD-L1* and CD8+ T cells in the CTNNB1 mutation and CTNNB1 wild type samples. **(I)** Boxplots of the expression of *CCL19* in the CTNNB1-Mutation group and CTNNB1-Wild type group in TCGA LIHC samples. **(J)** Boxplots of the expression of *CCL19* in the CTNNB1-Mutation group and CTNNB1-Wild type group in the GSE9829 dataset. **(K)** qPCR analysis of the expression of *CCL19* in the CTNNB1 wild-type HepG2 cells and CTNNB1 mutant Huh6 and SNU398 cells.

Overall, our classification system using the aging microenvironment-associated gene expression pattern across various cancer types divided the AME into the three subtypes that significantly correlated with OS in comparison with other LIHC classification approaches including Immune-Subtype, TME, and iCluster.

### The mutation of CTNNB1 inhibits the therapeutic effect of immune checkpoint inhibitors by suppressing the expression of CCL19

Previously, the hypothesis that LIHC “cold” tumors defined by Wnt/CTNNB1 mutation are those refractory to ICIs has been confirmed by Pinyol et al. (32). Immunotherapies in patients with “hot” tumors that were characterized by the presence of tumor-infiltrating lymphocytes (TILs) were more likely to be efficacious than in patients with “cold” tumors (36–38). About 30% of patients’ enrichment in *CTNNB1* mutation showed exclusion of TILs (36, 37). Consequently, tumors within the LIHC Immune Exclusion subtype might represent those with innate resistance to anti-PD1/PD-L1 or similar therapies. Therefore, it is necessary for us to explore the causes of immune checkpoint resistance in patients with *CTNNB1* mutation. To check whether the mutation of *CTNNB1* can affect the expression of immune checkpoints, we divided LIHC patients into *CTNNB1* mutated group and *CTNNB1* wild-type group. Then, by applying Wilcoxon test, we find that a large number of immune checkpoints were significantly differentially expressed between these two groups (**Figure 5A**). The same phenomenon was also verified in the GSE9829 dataset (**Figure 5B**). As shown in **Figure 2G**, the proportion of patients with *CTNNB1* mutation in the AME1 subtype was significantly higher than that of the AME2 subtype and AME3 subtype. Thus, the mRNA expression profile based on aging-related genes between the AME1 and other subtypes AME_O_ (O={2,3}) was compared by using limma package. |LogFC| >0.5 and FDR <0.05 were used as the criteria for screening differentially expressed aging-related mRNAs. Among the identified differentially expressed mRNAs, 87 downregulated mRNAs and 26 upregulated mRNAs in the AME1 subtype were selected as candidate markers (**Supplementary Figure S2A**). By applying Spearman’s correlation coefficient, we finally screened 11 aging-related genes that have a strong relationship with the activation of T or B cells (|Cor| >0.4 and P < 0.05), including *ZAP70*, *PIK3CD*, *PRKCQ*, *PRKCB*, *TNFSF13B*, *BTK*, *SYK*, *TNFRSF13C*, *CAMK4*, *CCL19*, and *TNFRSF11A* (**Figure 5C**). Interestingly, the expression of these genes was significantly restrained in the AME1 subtype. By using Wilcoxon test, *PRKCB* was excluded because it was not significantly differentially expressed between wild-type and mutated *CTNNB1* AME1 samples.

By comparing the correlation between the 10 screened genes and immune checkpoints (*PD-1*/*PD-L1*) in the *CTNNB1* mutated group and the wild type in AME1 patients (**Figures 5D, E**), we found that the correlation between these genes and immune checkpoints (*PD-1*/*PD-L1*) in the mutant group notably increased (**Supplementary Figures S3A**, **S3B**). Especially, the correlation of *CCL19*, *TNFRSF11A*, *TNFSF13B*, and *SYK* with immune checkpoints (*PD-1*/*PD-L1*) in the *CTNNB1* mutant group was significantly higher than that in the *CTNNB1* wild type (Cor_mut_-Cor_wild_ >0.1 and P < 0.05). Interestingly, the correlation between *CCL19* and *PD-L1* was not significant in the *CTNNB1* wild type (ρ = 0.0435, P = 0.607), whereas in the *CTNNB1* mutant group, the correlation between *CCL19* and *PD-L1* was significant (ρ = 0.254, P = 0.00339) (**Figures 5F, G**). Then, we investigated the correlation between the expression of these aging-related genes and CD8+ T cells (**Supplementary Figure S3C**). Consistent with previous results, the correlation between *CCL19* and CD8+ T cells was significantly higher in the mutant group than that in the *CTNNB1* wild-type group (ρmut = 0.681, P < 0.001; ρwild = 0.458, P < 0.001) (**Figure 5H**). Moreover, the expression of *CCL19* was significantly decreased in the *CTNNB1* mutation group (**Figure 5I**). We also found the same tendency in the external validation dataset: GSE9829 (**Figure 5J**). In addition, qPCR analysis was employed to verify this tendency. We found that the expression of *CCL19* in CTNNB1 wild-type hepatocellular carcinoma cells (HepG2 cells) is higher than that in CTNNB1 mutant cells (Huh6 and SNU398 cells) (**Figure 5K**, **Supplementary Table S2**). It has been reported that CCL19, as a chemokine, can promote the release of cytokines by CD8+ T cells by binding to its receptor CCR7, thereby inhibiting the proliferation, migration, and invasion of tumor cells (39). These results suggested that CTNNB1 mutation may inhibit the activation of immune cells by inhibiting the expression of CCL19, leading to the “immune cold” phenotype. Moreover, the correlation between the expression of *CCL19* and immune checkpoints (*PD-1/PD-L1*) in patients with *CTNNB1* mutation was significantly increased, which may also explain why AME1 subtype patients are resistant to anti-PD1/PD-L1 or similar therapies.

### The specific activation of mTOR is responsible for the poor prognosis of AME3 subtypes

**Figure 6:**
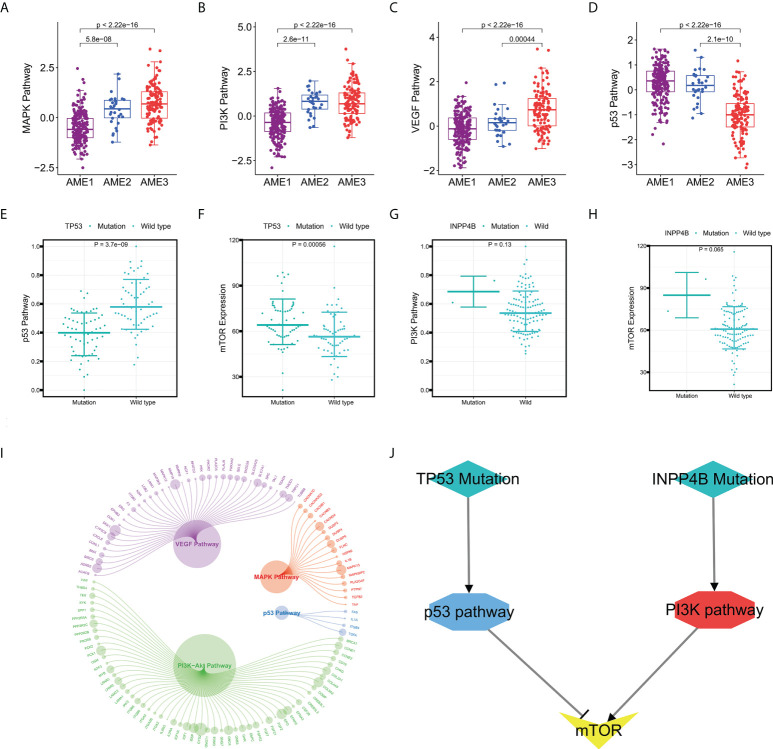
Identification of the differences in pathway activity among the three subtypes and their underlying mechanism. **(A–D)** The difference in pathway activity calculated by PROGENy among the three subtypes. **(E)** Boxplots of the p53 pathway activity in the TP53-Mutation group and TP53-Wild type group. **(F)** Boxplots of the mTOR pathway activity in the TP53-Mutation group and TP53-Wild type group. **(G)** Boxplots of the PI3K pathway activity in the INPP4B-Mutation group and INPP4B-Wild type group. **(H)** Boxplots of the mTOR pathway activity in the INPP4B-Mutation group and INPP4B-Wild type group. **(I)** Differentially expressed genes in the MAPK, PI3K, VEGF, and p53 pathways between AME3 and the other subtypes. **(J)** Regulatory axes for specific activation of mTOR.

To explore the potential reasons for the poor prognosis in patients with the AME3 subtype, we calculated the activity of 14 cancer-related signaling pathways, including the Androgen, Estrogen, EGFR, Hypoxia, JAK-STAT, MAPK, NFκB, PI3K, p53, TGFβ, TNFα, TRAIL, VEGF, and WNT pathways, which are available in the R package “PROGENy” (21) (**Supplementary Table S1**). By Kruskal–Wallis test, we identified significant differential expression among the three AME subtypes in all 14 pathways, among which the MAPK, PI3K, and VEGF pathways were specifically activated in the AME3 subtype, whereas the p53 pathway was specifically suppressed (**Figures 6A–D**). The percentage of tumors that carry TP53 mutation as one of the suppressors of the p53 signaling pathway (40) was significantly higher in the AME1 subtype than those in the AME2 and AME3 subtypes (**Figure 2G**). As expected, the p53 pathway was significantly suppressed in the TP53 mutation group (**Figure 6E**). Meanwhile, mTOR, a downstream molecule of the p53 pathway (41), was significantly activated in the TP53 mutation group (**Figure 6F**). These results suggested that the TP53 mutation may inhibit the activity of the p53 pathway, which promotes the expression of mTOR and then contributes to the proliferation, migration, and invasion of tumor cells.

In addition, we found that the PI3K pathway, another activator of mTOR, was specifically activated in the AME3 subtype (**Figure 6B**). To explore the reason for the specific activation of the PI3K signaling pathway, we investigated the relationship between the *INPP4B* mutation and the PI3K signaling pathway (42). As shown in **Figure 6G**, the activity of the PI3K signaling pathway and the expression of mTOR were significantly enhanced (**Figure 6H**), suggesting that the *INPP4B* mutation may promote the activation of the PI3K signaling pathway, thereby activating the expression of mTOR. Furthermore, we also computed the differentially expressed genes in these pathways including the MAPK, PI3K, VEGF, and p53 pathways between AME3 and other subtypes (**Figure 6I**). Collectively, we propose that specific activation of mTOR is responsible for the poor prognosis of AME3 subtypes; importantly, we uncovered several regulatory axes, including TP53/p53/mTOR and INPP4B/PI3K/mTOR (**Figure 6J**), providing clues for the treatment of the AME3 subtype.

### Establishment and evaluation of an aging-related gene signature in LIHC

In order to investigate the prognostic effects of these aging-related mRNAs, we established aging-related genes by applying LASSO and multiple Cox regression analysis. Different from the traditional methods, we adopted multiple iterations (times = 500) to avoid the contingency caused by the random error of LASSO regression. This process can be divided into five steps: 1) Aging-related genes associated with prognosis were screened out by univariate Cox regression, and those with low variance were removed (Var <5); 2) The selected aging-related genes were dimensionally reduced by LASSO regression, repeated 500 times, and the total number of occurrences of each gene was counted; 3) Genes, total occurrences, number of more than 100 times were retained; 4) The model with the maximum AUC value was selected as the final prognostic model through multiple repetitions; 5) Calculate the prognostic signature for each sample.

**Figure 7:**
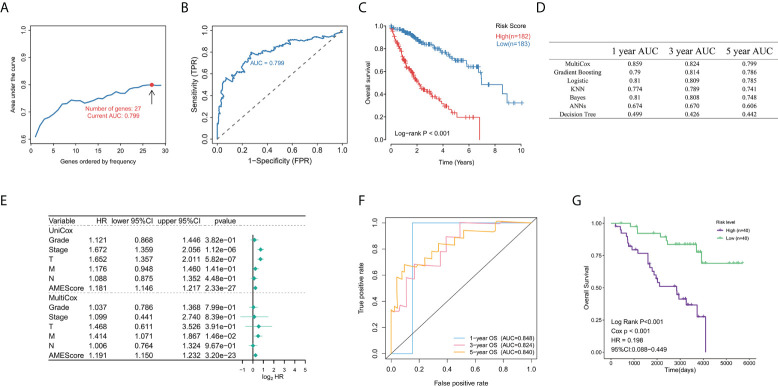
Establishment and evaluation of an aging-related gene signature. **(A)** Multiple LASSO regression and Cox regression were used to define the optimal model. **(B)** Time-dependent ROC curve analysis of the model in LIHC at 1, 3, and 5 years. **(C)** Kaplan–Meier estimates of overall survival of patients with High Risk and Low Risk in TCGA LIHC samples. **(D)** The AUC of models established by multiple algorithms. **(E)** Univariate Cox regression analysis for identifying our model was significantly correlated with prognosis, and multivariate Cox regression analysis for identifying our model could be used as an independent prognostic factor. **(F)** Time-dependent ROC curve analysis of the validation data (GSE20140). **(G)** Kaplan–Meier estimates of overall survival of patients with High Risk and Low Risk in the validation data (GSE20140).

Through this process, we finally selected 27 genes to establish a model corresponding to the maximum AUC (5-year AUC = 0.799) (**Figures 7A, B**), including *AKT2*, *ATG101*, *BMI1*, *BMP6*, *CCNA2*, *CD40LG*, *COQ7*, *CXCL8*, *DLAT*, *EEF1E1*, *EIF4E*, *GCLM*, *HDAC1*, *HDAC2*, *HTRA2*, *IL7R*, *MAPT*, *MMP1*, *PIK3R1*, *PON1*, *PPARGC1A*, *SOCS2*, *TFDP1*, *TRAF3*, *ZAP70*, *UBB*, and *JUND*. The results from the K-M analysis suggested that patients with high risk have lower OS rate than patients with low risk in LIHC patients in TCGA (P < 0.05) (**Figure 7C**). In order to verify the advantage of this model, we applied these 29 genes to construct multiple models including Grading Boosting (1-year AUC = 0.790; 3-year AUC = 0.814; 5-year AUC = 0.786), Logistic Regression (1-year AUC = 0.810; 3-year AUC = 0.809; 5-year AUC = 0.785), K-Nearest Neighbor (1-year AUC = 0.774; 3-year AUC = 0.789; 5-year AUC = 0.741), Bayesian (1-year AUC = 0.810; 3-year AUC = 0.808; 5-year AUC = 0.74), Artificial Neutral Network (1-year AUC = 0.674; 3-year AUC = 0.670; 5-year AUC = 0.606), and Decision Tree (1-year AUC = 0.499; 3-year AUC = 0.426; 5-year AUC = 0.44), and we found that our model has dominant credibility and predictive value (**Figure 7D**). Univariate Cox regression results indicated that our model was significantly correlated with prognosis, and multivariate Cox regression results suggested that our model could be used as an independent prognostic factor that was independent from other clinical factors, including Stage, T, N, and M (**Figure 7E**). In addition, a cross-platform validation from GSE20140 was involved to verify the accuracy of the model. A total of 20 genes were included in the list of 29 genes in the GSE20140 validation set, and the risk model was established through multivariate Cox regression. The ROC curve prompted that this model had dominant credibility and predictive value (1-year AUC = 0.848; 3-year AUC = 0.824; 5-year AUC = 0.840) (**Figure 7F**). By constructing K-M analysis, this model was proven to have a great prognostic credibility (P < 0.05) (**Figure 7G**).

We further investigated molecular events at single-cell resolution by comparing high- and low-risk tumors categorized by our aging gene-based model. We annotated seven major cell types for 10 primary liver tumor tissues from the GSE149614 dataset using biomarkers ACTA2 (Fibroblast cells), CD68 (Myeloid cells), ALB (Hepatocyte cells), CD3D (T cells), JCHAIN (Plasmablast cells), MS4A1 (B cells), and PECAM1 (Endothelial cells) (**Supplementary Figures S4A**, **S4B**). By applying the AUCell (43) algorithm, we calculated the score of each cell through these 27 gene features that comprised our model and determined HCC01T, HCC06T, HCC07T, and HCC09T samples as high-risk samples and HCC02T and HCC04T as low-risk samples (**Supplementary Figure S4C**). To characterize intercellular interactions in high- and low-risk groups, we inferred putative cell-to-cell interactions based on ligand–receptor signaling using CellChat (44). Interestingly, we observed globally enhanced intercellular interactions for the low-risk group (**Supplementary Figures S4D**, **S4E**). In addition, we found that CD46 signaling network was strengthened in the low-risk group, which suggested that it may play an important role in the process of inhibiting tumor progression (**Supplementary Figure S4F**). These results confirmed that aging-related genes play a crucial role in affecting the tumor microenvironment.

Overall, aging-related genes have good efficacy in predicting the prognosis of patients with LIHC, and they can serve as regulators in the tumor microenvironment.

### The activation of the PI3K/AKT/mTOR signaling pathway is related to the progression of liver cancer

**Figure 8:**
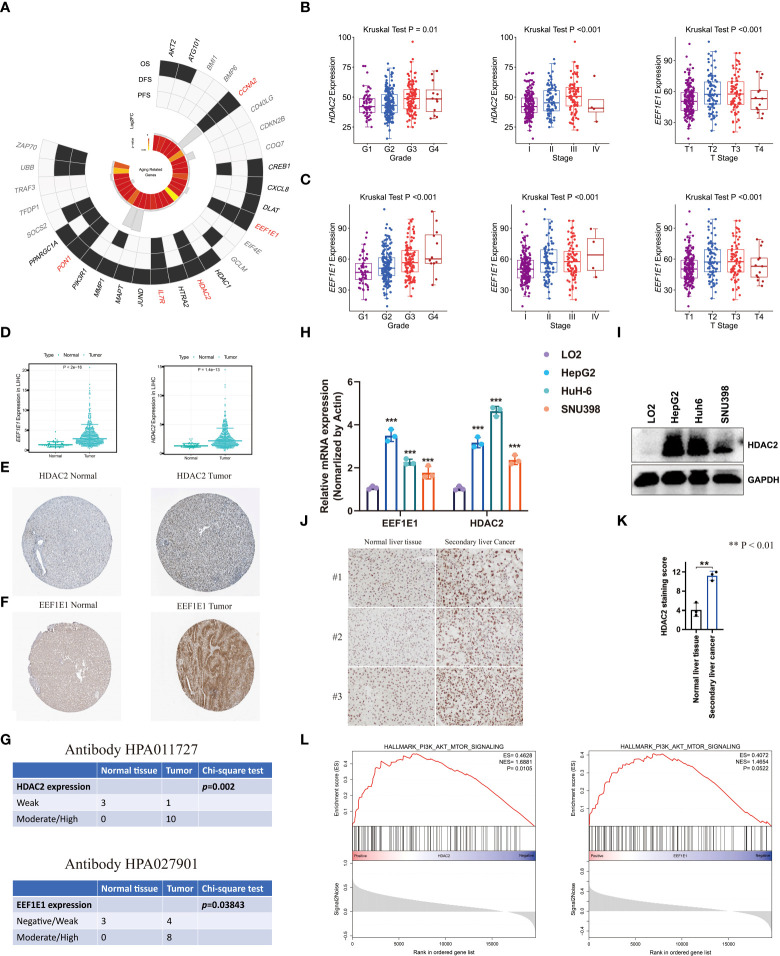
Identification of aging-related gene hubs associated with liver cancer disease progression. **(A)** Five aging-related genes were selected by using Kaplan–Meier estimates of overall survival, progression-free survival, and disease-free survival. **(B, C)** Two aging-related gene hubs were selected by using the correlation between aging-related genes and clinical feature. **(D)** The expression level of *HDAC2* and *EEF1E1* in adjacent normal liver and LIHC samples. **(E, F)** The protein expression level of HDAC2 and EEF1E1 using the HPA database. **(G)** Chi-square test of the significance between the number of normal tissue samples and hepatocellular carcinoma samples in protein expression level of HDAC2 and EEFIE1. **(H)** qPCR analysis of EEF1E1 or HDAC2 expression in the hepatocyte line LO2 and hepatocellular carcinoma cell lines HepG2, Huh6, and SNU398. The experiment was performed three times with consistent results; representative results from one set of experiments are shown. **P < 0.01; ***P < 0.001.**(I)** Immunoblot analysis of HDAC2 levels in the hepatocyte line LO2 and hepatocellular carcinoma cell lines HepG2, Huh6, and SNU398. Experiment was performed three times with consistent results; representative image is shown. **(J)** IHC analysis of HDAC2 in normal liver tissues and secondary liver cancer tissues. **(K)** HDAC2 staining score in normal liver tissues and secondary liver cancer tissues. **(L)** GSEA was employed to explore the biological function of these aging-related genes (*HDAC2* and *EEF1E1*) in the progression of LIHC.

In order to explore hub aging-related genes associated with liver cancer disease progression, we first screened aging-related genes that were significantly associated with survival in the model. The results from the K-M analysis suggested that CCNA2, *EEF1E1*, *HDAC2*, *IL7R*, and *PON1* were significantly associated with OS, progression-free survival, and disease-free survival (P < 0.05, **Figure 8A**). To define the correlation between five aging-related genes and clinical feature, Kruskal–Wallis test was involved. The result suggested that *EEF1E1* and *HDAC2* were associated with the progression of tumors where they show significant correlation with grade, stage, and T stage (P < 0.05; **Figures 8B, C**), and the expression levels of *EEF1E1* and *HDAC2* were significantly higher in tumor tissue than in normal tissue (**Figure 8D**). In addition, using qPCR, we found that the mRNA expression of *EEF1E1* and *HDAC2* in hepatocellular carcinoma cells (HepG2, Huh6, and SNU398) are higher than those in normal hepatocytes (LO2) (**Figure 8H**, **Supplementary Table S2**).

Using the HPA database, we found that the protein expression level of EEF1E1 and HDAC2 were higher in liver cancer while lower in normal liver (**Figures 8E–G**). By using the chi-square test, we found a significant difference in the protein expression level of normal tissue samples and hepatocellular carcinoma samples in the HPA database (**Figure 8G**). Using qPCR and Western blotting, we found that the mRNA and protein levels of HDAC2 in hepatocellular carcinoma cells (HepG2, Huh6, and SNU398) are higher than those in normal hepatocytes (LO2) (**Figures 8H, I**). We also found an mRNA level increase in *EEF1A1* expression in LIHC cancer cells (**Figure 8H**). Interestingly, we also found that HDAC2 levels in secondary liver cancer tissues are higher than those in normal liver tissues, which further supports that HDAC2 plays an important role in the progression of liver cancer (**Figures 8J, K**).

To explore the biological function of these aging-related genes (*EEF1E1* and *HDAC2*) in the progression of LIHC, we performed GSEA based on TCGA cohort. Enrichment result indicated that the PI3K/AKT/mTOR signaling pathway can be activated by EEF1E1 and HDAC2 (P < 0.05, **Figure 8L**), which is consistent with previous results. The PI3K/AKT/mTOR pathway has been proven that it is aberrantly hyperactivated in many types of cancer that has a strong relationship with poor clinical prognosis. In the tumor microenvironment, the PI3K/AKT/mTOR pathway plays an important role in promoting the proliferation and metastasis of tumor while strongly inhibiting the antitumor immune response (45).

Interestingly, this result indicated that the PI3K/AKT/mTOR pathway may be a potential pathway that aging-related genes converge on to liver tumor progression.

## Discussion

LIHC is the fifth leading cause of malignant cancer and the third most common cause of cancer-related death worldwide (1). Recent studies have shown that aging-related genes can serve as a risk factor in LIHC and can be used to predict the prognosis of patients (46, 47). However, the mechanism behind aging-related genes affecting the prognosis of LIHC patients and the effect of aging-related genes in immunotherapy are still unclear.

Here, we comprehensively characterized aging-related clinical and molecular features in LIHC by an integrated analysis of public datasets. Based on aging-related genes, we established three prognosis-distinct aging subtypes in LIHC, including “immune exclusion” AME1, “immune infiltration” AME2, and the intermediate AME3 subtypes. We identified a high level of *CTNNB1* mutations in AME1, which by previous knowledge causes insensitivity to immunotherapies. We identified CCL19 as a potential key gene downstream of CTNNB1 to convey the immunosuppressive effect. In addition, we uncovered activation of the oncogenic PI3K/AKT/mTOR pathway in AME3 by multiple aging-related genes including *EEF1E1* and *HDAC2*. Our finding suggested that the aging microenvironment could promote liver tumor progression and treatment sensitivity *via* modulating immune profiles and oncogenic signaling, which potentially could lead to better prognosis and treatment selection.

Our AME subtypes are characterized by the different levels of immune cell infiltration, which is largely consistent with the conventional immune subtypes, yet performed better in prognosis. Among them, the AME2 subtype is characterized by numerous TILs and high PD-L1 expression, which suggest that the AME2 subtype belongs to the tumor microenvironment immune type I (TMIT-I) with optimal benefit for ICIs (48). The AME1 subtype was characterized by specific inhibition of immune infiltrating cells. Almost all immune infiltrating cells were suppressed. Meanwhile, the expression of immune checkpoints was restrained in the AME1 subtype. To date, ICIs, such as nivolumab (PD-1 inhibitor), have been approved by the Food and Drug Administration (FDA) for the treatment of several cancers (49, 50). Yet, the responses of patients to ICI therapies vary greatly, with some patients achieving complete remission and others showing continuous progression (51). Hence, our subtypes could assist in decision-making for the ICI treatments of LIHC.

Previous studies have shown that approximately 30% of LIHC patients can be defined as “Immune Exclusion class,” characterized by *CTNNB1* mutation and insensitive to ICIs such as anti-*PD1*/*PD-L1* or similar therapies (36, 37). We found that the frequency of *CTNNB1* mutation was significantly higher in the AME1 subtype than that in the other two subtypes. It has been proven that LIHC patients with *CTNNB1* mutation may be insensitive to immunotherapy (32). However, the mechanism behind this phenomenon is still unclear. An increasing number of studies have found that certain key factors influence the progression of tumors in an age-dependent manner, for example, AKR1B10, which is highly expressed in liver cancer tissues and is positively correlated with the level of alpha fetoprotein and the proportion of liver cell steatosis, was identified as a crucial gene in the increase of carcinogenesis with age (52, 53). Hence, it is of great significance to study the role of aging-related genes in tumor progression. In our study, we found that the expression of *CCL19* was significantly decreased in the *CTNNB1* mutant group. CCL19 was reported previously to recruit Dendritic cells (DCs) and T cells in paracancerous tissues to tumor tissues (54). In addition, in mouse CAR-T cells, *IL-7* and *CCL19* expressions have been demonstrated to improve T-cell infiltration and Chimeric Antigen Receptor T (CAR-T) cell survival in mouse tumors (55). It has been uncovered that compared with conventional *CD19* CAR-T cells, the combination of *IL7* and *CCL19* can promote the infiltration, accumulation, and survival of CAR-T cells in lymphoma tissues, further enhancing the antitumor effect of conventional CAR-T cells (54). This suggested that CTNNB1 mutation may inhibit the expression of immune checkpoints and affect the immune response of patients by inhibiting the expression of *CCL19* (39), which will provide a potential target for immunotherapy.

Interestingly, the MAPK, PI3K, and VEGF pathways were activated in poor prognostic subtype AME3 and may contribute to tumorigenesis. However, the p53 pathway was specifically restrained in the AME3 subtype. Inactivation of the transcription factor p53, through either direct mutation or aberrations in one of its many regulatory pathways, is a hallmark of almost every tumor (56). In addition, in the tumor microenvironment, the PI3K pathway plays an important role in promoting the proliferation and metastasis of tumor while strongly inhibiting the antitumor immune response (45). We identified INPP4B as an upstream regulator of the PI3K and p53 pathways. INPP4B is a tumor suppressor gene that inhibits the PI3K signaling pathway (42). We found that the PI3K pathway activity was activated in the INPP4B mutant group. However, the mutation frequency of INPP4B in AME3 subtype was not significantly different from that of the other two subtypes, which may be influenced by the small mutant number (size = 2). In addition, we found that TP53, the most commonly mutated gene in human cancer (57), mutated at a significantly higher frequency in the AME3 subgroup than in the AME1 and AME2 subtypes. Meanwhile, the p53 pathway was significantly inhibited in the TP53 mutant group. mTOR, as a common downstream regulator of the p53 pathway and PI3K pathway (4, 40), was significantly activated in the TP53 mutation group and INPP4B mutation group. Therefore, our study suggests that TP53 and INPP4B may influence the expression of mTOR by influencing the PI3K and p53 pathways. Consequently, the activation of two oncogenic axes, TP53/p53/mTOR, INPP4B/PI3K/mTOR, may explain the reason why the AME3 subtype had the worst survival. These results will help clinicians in ranking the tumor and therapies with more information. Specifically, clinicians can assign patients to specific AME subtypes based on their immune and mutation characteristics. For example, if a patient was assigned to the AME1 subtype, clinicians need to be aware that these patients are not suitable for immunotherapy. Furthermore, if a patient has a high-frequency mutation rate of TP53 or INPP4B, then clinicians need to notice whether this patient is accompanied by specific activation of the expression of mTOR, which is usually the worst case.

To verify the conservation of our subtypes, we classified multiple external LIHC validation sets using supervised clustering and verified the classification method in multiple cancers. In a previous study, it has been uncovered that an increased senescence score was associated with increasing age and higher malignancy and is somewhat associated with poor prognosis (46). In addition, the tumor microenvironment associated features have been employed to establish prognostic models with good results (58). Hence, we also established a robust aging-related model based on age-related genes to predict the prognosis of LIHC patients. The gene selection process in our model adopts multiple iterations (times = 500) to improve the contingency in the gene selection process and avoid the random error of LASSO regression; furthermore, the modeling method was selected from a variety of methods based on credibility and predictive values. Our model predicted survival with an AUC >0.799, which is superior to the previous LASSO regression-based prognostic model in LIHC (AUC <0.78) (47).

We also explore hub aging-related genes and potential pathways associated with liver cancer disease progression. We defined five hub aging-related genes including *CCNA2*, *EEF1E1*, *HDAC2*, *IL7R*, and *PON1* by using K-M analysis and found that *EEF1E1* and *HDAC2* were associated with the progression of tumors where they showed significant correlation with Grade, Stage, and T stage. *EEF1E1A* is a novel LIHC prognosis predictor and associated with immune infiltration (59). A recent study showed that *HDAC2* can bind to miR-503-5p and target *CXCL10*, thus promoting the progression of esophageal squamous cell carcinoma (60). These hub genes were further shown to correlate positively with the PI3K/AKT/mTOR signal pathway molecules. The PI3K/AKT/mTOR pathway is commonly hyperactivated in many types of cancer and correlates with poor clinical prognosis (45). Consistent with previous conclusions, the activation of the PI3K/AKT/mTOR pathway may be downstream of the hub genes that we identified in our study to promote the disease progression of LIHC patients.

In conclusion, we developed an effective strategy for classifying LIHC from a new perspective and revealed the link between the aging microenvironment and tumor immunity as well as tumor mutations. Meanwhile, the mechanism of CTNNB1 mutation affecting immune insensitivity in LIHC patients was also uncovered. These results provide clues for predicting the prognosis of LIHC and potential therapeutic targets, which may contribute to the diagnosis and treatment of LIHC.

## Data availability statement

The original contributions presented in the study are included in the article/**Supplementary Material**. Further inquiries can be directed to the corresponding authors.

## Author contributions

GL, CT, TH, and YP designed this project; YP and TH performed the bioinformatics analysis. TH and WS constructed the experiment. TH and YP wrote the manuscript. GL and CT revised the manuscript and performed the data review. GL supervised the project, established the collaboration, and provided funding support for the project. All authors read and approved the manuscript.

## Funding

This work was supported by the research grants from the National Natural Science Foundation of China (82003190).

## Conflict of interest

The authors declare that the research was conducted in the absence of any commercial or financial relationships that could be construed as a potential conflict of interest.

## Publisher’s note

All claims expressed in this article are solely those of the authors and do not necessarily represent those of their affiliated organizations, or those of the publisher, the editors and the reviewers. Any product that may be evaluated in this article, or claim that may be made by its manufacturer, is not guaranteed or endorsed by the publisher.
