# Supplementary figures and images for: Aging-related features predict prognosis and immunotherapy efficacy in hepatocellular carcinoma
Source: Front Immunol. 2022 Sep 15;13:951459. doi: 10.3389/fimmu.2022.951459 (PMC9521435; doi:10.3389/fimmu.2022.951459)

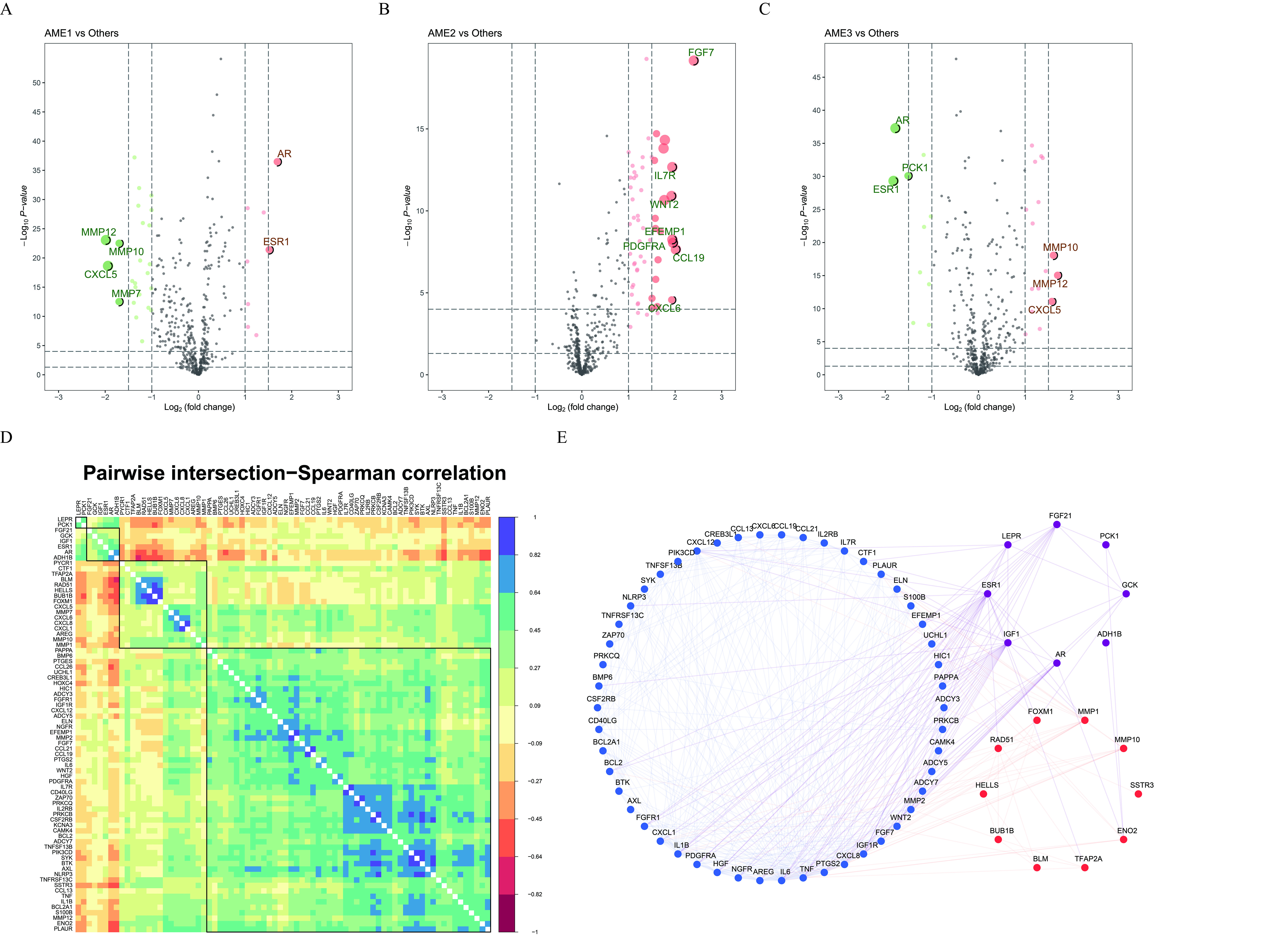

Supplement: Supplementary file 2 [file Image_2.jpeg]

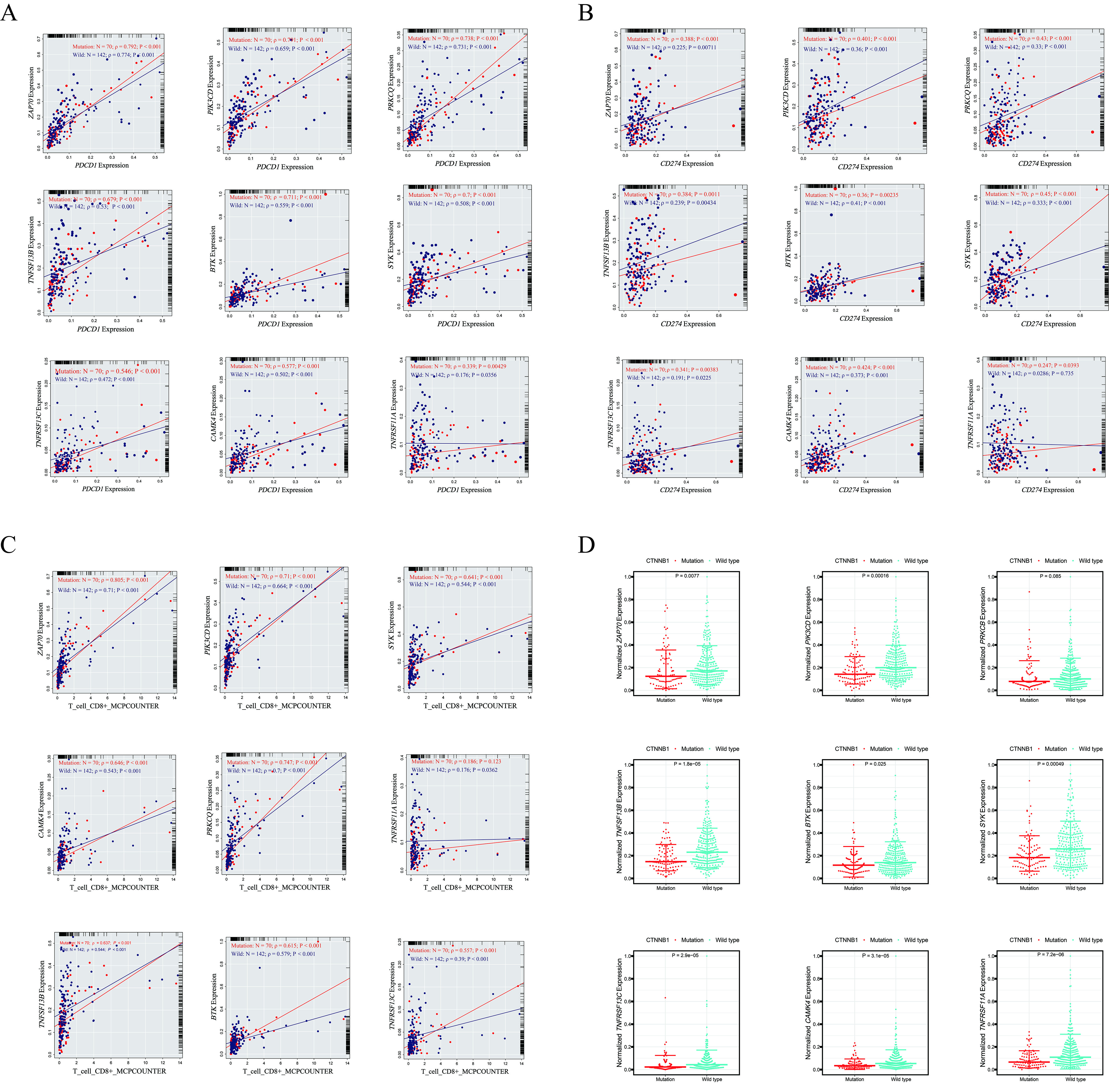

Supplement: Supplementary file 3 [file Image_3.jpeg]

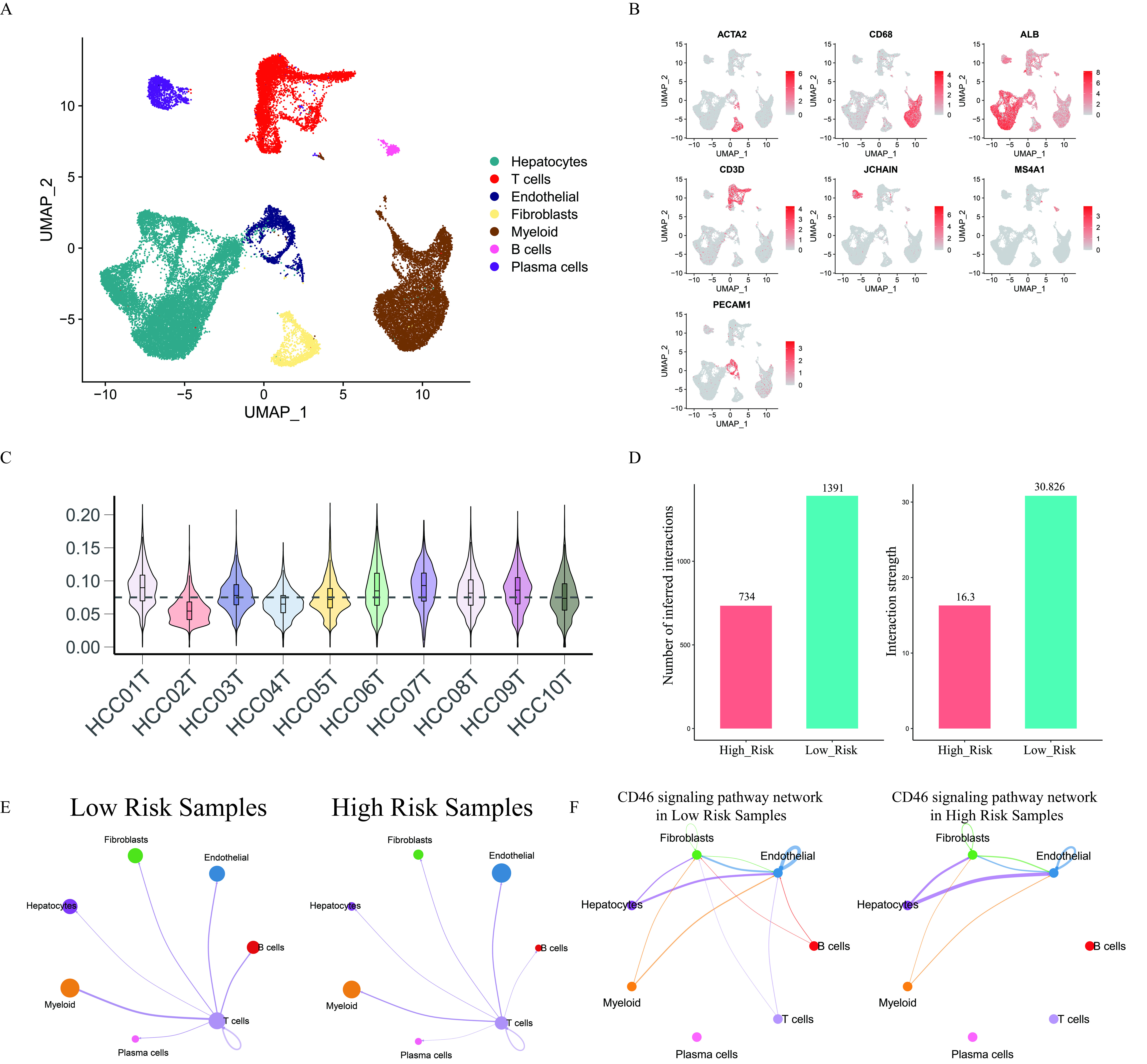

Supplement: Supplementary file 4 [file Image_4.jpeg]
