# Supplementary material for: Aging-related features predict prognosis and immunotherapy efficacy in hepatocellular carcinoma
Source: Front Immunol. 2022 Sep 15;13:951459. doi: 10.3389/fimmu.2022.951459 (PMC9521435; doi:10.3389/fimmu.2022.951459)
Supplement: Supplementary file 7 [file Table_3.doc]

Supplementary Table S3 Primers for RT-qPCR

| **Gene** | **Forward Primer** | **Reverse Primer** |
| --- | --- | --- |
| ***EEF1E1*** | CCCTGGGACTGAGTAAGGGG | GTTGGCTTGCTTGACTAGATGA |
| ***HDAC2*** | ATGGCGTACAGTCAAGGAGG | TGCGGATTCTATGAGGCTTCA |
| ***Actin*** | GGGACCTGACTGACTACCTC | TCATACTCCTGCTTGCTGAT |

*EEF1E1, Eukaryotic Translation Elongation Factor 1 Epsilon 1; HDAC2, Histone Deacetylase 2*
